# Undocumented adult migrants in Sweden: mental health and associated factors

**DOI:** 10.1186/s12889-018-6294-8

**Published:** 2018-12-12

**Authors:** Lena M.C. Andersson, Anders Hjern, Henry Ascher

**Affiliations:** 1 Department of Social Work, University of Gothenburg, Sprängkullsgatan 23, PO Box 720, SE- 405 30 Gothenburg, Sweden; 2 Clinical Epidemiology, Department of Medicine, Karolinska Institute, Stockholm, Sweden; 3 Centre for Health Equity Studies (CHESS), Karolinska Institute and Stockholm university, Stockholm, Sweden; 4 Department of Public Health and Community Medicine, Sahlgrenska Academy, University of Gothenburg, P.O. Box 453, SE - 405 30 Gothenburg, Sweden; 5 Department of Research and Development, Angered Hospital, Gothenburg, Sweden

**Keywords:** Undocumented migrants, Mental health, Sweden

## Abstract

**Background:**

Undocumented migrants (UMs) in Europe constitute a heterogeneous group. They are typically in a vulnerable and marginalised situation, since most of them have exhausted their options for gaining asylum and protection from war and persecution, many are traumatised and fear disclosure and deportation, and they typically lack basic social security. The present study investigates living conditions, access to human rights and mental health of UMs living in Sweden.

**Methods:**

A cross-sectional study with adult UMs was performed in the three largest cities in Sweden in 2014–2016. Sampling was done via informal networks. A socioeconomic questionnaire was constructed, and psychiatric symptoms were screened for using Beck’s Depression Inventory II, Beck’s Anxiety Inventory and the PTSD Checklist (PCL) for civilians. Trained field workers conducted the interviews. Descriptive statistics, chi-square tests and logistic regression models were used.

**Results:**

A total number of 104 individuals participated. Preliminary findings show that 68% of respondents were suffering from either moderate or severe anxiety, 71% from either moderate or severe depression and 58% from PTSD. No statistically significant gender differences occurred, but age was statistically significant in relation to anxiety and depression. The majority feared returning to their country of origin, for political reasons, due to war in progress there and/or because they belonged to a minority and feared harassment. Almost all had an unstable housing situation and were often forced to move. Fifty-seven percent experienced food insecurity.

**Conclusion:**

The psychosocial situation among UMs in Sweden, in addition to insecure living conditions without a guarantee of basic needs being met is stressful, and many UMs live in constant fear of disclosure and deportation, all of which has a detrimental effect of the mental health. It is important to understand both associated risk factors for ill-health and coping strategies in this vulnerable population in order try to reduce ongoing stress.

## Background

People living in a country without a residence permit are often labelled as undocumented or irregular. This group is not homogenous: some are tourists, students or labour migrants overstaying their visas or residence permits. Others may have entered the country without permission, most often due to poverty and with the purpose to improving this situation. A third group consists of refugees who have entered the country legally in order to seek asylum but whose asylum claims have finally been rejected [1]. Although these subgroups are also heterogeneous, it can be expected that undocumented former asylum-seekers will make up a particularly vulnerable group [2]. Many of them are victims of war, violence and forced migration. Moreover, they may have experienced further trauma while fleeing and/or during a prolonged asylum process where they may experience their traumatic experiences being disapproved.

A common feature of persons in an undocumented situation is that they are ‘deportable’: if their status is disclosed they risk deportation to their country of origin. The consequences of this differ between the groups mentioned. For most tourists and students, they are probably relatively minor in relation to rejected asylum-seekers, for whom deportation may be regarded as posing a potential danger to life. The latter group in particular could thus be expected to live under heavy stress and in constant fear, often aggravated by previous traumatic experiences as well as social and economic hardship, all potentially increasing the risk of ill-health [3].

Undocumented migrants (UMs) in Sweden have traditionally mainly consisted of former asylum-seekers, although the number of EU citizens exceeding the permitted period of residence without a labour contract of three months has increased in recent years. Living as an UM in Sweden is complicated since access to basic services as well as welfare system requires the individual to have a unique ten-digit personal ID number. However, since 2013 new laws have been passed giving UM adults access to health care and dental care ‘that could not be deferred’, while children have full access to health care, dental care and schooling [4].

Despite their vulnerability described above, there is a lack of epidemiological data on UMs. In a European review, Woodward et al. [5] found that most published studies on UMs were qualitative. Sampling descriptions were often of poor quality, and only four studies used randomisation techniques. Most (80%) studies focused on access to health care, and many are clinical samples. Castañeda et al. [6] interviewed 183 UM patients who attended a health clinic that provided the bulk of health care for UMs in Berlin in 2004–2006 and 2008. The focus was on understanding how ‘illegality’ influenced UM experiences, medical treatment and convalescence. Results showed that living as an undocumented migrant was associated with health effects in various ways: it reduced the overall quality of care for mothers and infants, delayed the seeking of help, was associated with difficulties in accessing medication for patients with chronic illnesses and created difficulties in accessing care for acute health concerns. Furthermore, there was a lack of mental health care and there were high rates of generalised stress, anxiety that affected health in such a way that both patients and physicians referred to it as the ‘illegal syndrome’.

Schoevers et al. [7] interviewed 100 UM women in the Netherlands and found that 65% of them rated their health as ‘poor’. The majority were suffering from anxiety (78%), insomnia (75%), agitation (71%), depressed mood (64%), and nightmares (53%). In a German study by Kuhne et al. [8] of 96 UMs who attended a non-governmental organisation for medical counselling, many respondents rated their health-related quality of life as low. In addition, the authors conducted an ethnographic study and found that the three factors the respondents felt impeded their health most were socioeconomic conditions, experiences of criminalisation and late access to health care facilities. Other risk factors for mental illness among UMs were found in a scope literature review [9] where mental illness among UMs was associated with barriers to health care, exploitative work conditions, pregnant women being denied care, women being exposed to domestic violence [10], and, in relation to trafficking, being at risk of developing HIV/AIDS and STIs [11]. UMs described their experiences of living undocumented as ‘degrading’ and ‘unbelievable’ [12], living with a constant fear of deportation and anxiety over health care costs, experiences of isolation, social exclusion [10] and lack of social support [13].

Despite high numbers of asylum-seekers in Sweden, knowledge of health and living conditions among UMs is still scant, and there is a lack of population-based studies in particular [1]. With the exception of a few studies such as a study by Wahlgren et al. [14] investigating causes of deaths among UM, a study from Stockholm County Council [15], a recent study of UM women seeking perinatal care [16], two PhD theses [17, 18] and master’s theses [19, 20], findings on UMs stem from smaller studies performed by non-governmental organisations (NGOs) such as the Red Cross [21], Doctors in the world [22] and Doctors without borders [23]. These studies describe highly precarious living conditions. UMs in Sweden live in expensive housing with limited space, in temporary conditions and are confined to dangerous and poorly paid work in the informal sector [22–24]. In a study performed in 2005 [23] it was found that among 102 persons visiting an informal health clinic, almost 40% had no work and 45% had only occasional work. Most were underpaid, and worked in the informal sector in cleaning, construction, restaurants and shops. Of the respondents, 23 persons answered questions about mental health and half were suffering from anxiety and depression (Hopkins Symptom Checklist). Sixty-four per cent regarded their mental health as impaired during their time as undocumented migrants. As mentioned, this data is based on small samples of unclear representativeness [1].

In Europe, all countries have ratified the International Covenant on Economic, Social and Cultural Rights (ICESCR) [25] and the Charter of Fundamental Rights of the European Union [26]. These international conventions include binding commitments concerning the right to the highest attainable standard of physical and mental health. The practical meaning of the right to health is further developed in General Comment 14 [27]. The ICESCR also includes the right of the basic needs for life, such as access to essential food that is nutritionally adequate, shelter, housing and sanitation, and these rights apply regardless of whether the individual has legal status to stay in the country. Human rights are not an entitlement but, as the Office of United Nation High Commissioner for Human Rights (OHCHR) states [28], states still need to think how they shall guarantee that all people, citizens as well as non-citizens, have their human rights met in their social policies. In Sweden, UM have access to health care “that cannot be deferred”. However, assistance from the Social Services have varied, since the municipalities have interpreted the Social Service Act differently.

### Aim

The overall aim of this project; the Swedish Health Research on Undocumented Migrants project (SHERUM), is to describe the physical and mental health of UMs, their social and psychological functioning, the impact of pre-migration, migration and post-migration factors on their health, and to investigate how UMs assess their health service needs and what coping strategies they use to manage their situation. In this article, in which the methodology of the study is also presented in detail, we focus on mental health and present findings concerning the prevalence of and risk factors for anxiety, depression and post-traumatic stress syndrome among adult UMs in Sweden. This study is, to our knowledge, the most current and to date largest study on a non-patient UM population in Scandinavia.

## Methods

### Setting and study population

The data for this cross-sectional study was collected in the three largest cities in Sweden, Gothenburg, Stockholm and Malmö, during the period 2014–2016. Included in the study were persons over 18 years of age, defined as UMs because of any of the following: a) having applied for asylum and a residence permit but the application having been rejected and the decision having gained legal force; b) persons from outside the EU having overstayed in Sweden after their visa has expired; or c) persons having moved to Sweden without applying for a visa. Excluded from the study were migrants from EU countries who had overstayed their visa.

### Sampling of the study population

Because of the fear UM commonly have of authorities, especially the fear of being disclosed and deported, this study had to use unconventional methods to make contact with and recruit respondents. We therefore aimed at creating a convenience sample. The researchers contacted representatives of various informal networks that provide support to UMs in Stockholm, Gothenburg and Malmö to make contact with UMs. The network around the Rosengrenska clinic in Gothenburg, Doctors of the World in Stockholm and the Red Cross in Stockholm and Malmö, organisations that provide informal health care and financial support to UMs, were particularly important in providing contacts with informants, but churches and other supporting networks and organisations were also contacted. These networks provide a variety of services to UMs, serve as meeting places for social support and/or provide clothes, food and health services. The majority of respondents in our study did not visit the clinic due to physical or mental problems, but because of social activities and food distribution. Within these networks, persons that held a position of trust among the migrants, often a nurse or social worker, asked potential respondents if the researchers could contact them, informed them about the study and asked them to take part. When researchers/interviewers informed the UM about the study, a translator was present so that correct information could be given in the respondent’s own language. An information brochure was handed out, presenting the study in nine languages. In cases where no translator was available at the clinic, a later meeting was set up. Both authorised translators and volunteer translators were used at the clinics for this information. The written and oral information clearly stated that the researchers were not in any way associated with any authorities, that anonymity was strict and that participation in the study would not affect their asylum cases. When an UM accepted being interviewed, an agreement was made on time and place. Most interviews were done in churches and voluntary organisations. It was crucial that the respondent felt secure and safe with the time and setting. Psychological counselling was offered if the respondent needed it after the interview. About five respondents felt such a need. The final sample consisted of 104 UMs: 54 men and 50 women**.** In this paper, we present findings from those respondents who answered all questions on at least one of the three psychiatric scales, resulting in 88 individuals, 48 men and 40 women.

### Measurements

#### The questionnaire

An extensive questionnaire concerning living conditions and health was developed in the project in collaboration with a reference group consisting of four persons who were, or recently had been, in an undocumented situation. Items used in other studies in the field of migration and/or health were used together with items developed specifically to meet the aims of this study. The questionnaire consisted of 112 multiple-choice questions concerning the following topics: background, family and children, access to food, living conditions, education, work and income, maternal care, physical and mental health, access to care and experiences of care, medicine, barriers to care, social support and social networks, traumatic events in Sweden, experiences of violence in Sweden, strategies when undocumented, views on returning to the country of origin. Open-ended questions were also used. After piloting of the questionnaire, the following sections were withdrawn from the questionnaire since we realised that it was too long and potentially exhausting, and were explored in qualitative interviews instead: child health and living conditions among children, social support and social networks, traumatic events in Sweden, and strategies when undocumented.

In addition to the questionnaire, three psychiatric instruments were included in the study to screen for the respondent’s mental health.

*Beck’s Depression Inventory* (BDI-II) is a scale that has been widely used and has been validated in many populations and cultural groups. This instrument consists of 21-item multiple choice questions based on DSM-IV criteria for depressive disorders. The questions concern *the past two weeks*, and responses are rated on a 4-point Likert scale, ranging from 0 (not at all) to 3 (severely). The 21 items are combined into a single sum score, ranging from 0 to 63. A sum score of 0–13 is considered to be a minimal score of depressive symptoms, 14–19 is considered a mild, 20–28 a moderate, and a sum score of 29–63 a severe range of depressive symptoms [29].

*Beck’s Anxiety Inventory (BAI)* is a similar 21-item multiple choice instrument that assesses anxiety symptoms with a focus on somatic symptoms of anxiety that have occurred in *the last week* [30]. Responses are rated on a 4-point Likert scale and range from 0 (not at all) to 3 (severely). The BAI is developed to measure anxiety that is relatively independent of depression. The total score ranges from 0 to 63. The following interpretation of scores is suggested: 0–9, normal or no anxiety; 10–18, mild to moderate; 19–29, moderate to severe; and 30–63, severe anxiety [31].

*PCL- 5* – is a screening tool to measure post-traumatic stress disorder (PTSD). It measures symptoms according to DSM-5 during *the past month*. Total scores range 0–80, and suggested cut off for a PTSD diagnosis is 38 [32]. The scale consists of an introductory text that gives examples of several stressful experiences, or likewise that may have happened to the respondent. If so, five questions about the worst event follow. If there are several traumatic events, the respondent is asked to think about the worst when answering the following 20 multiple-choice questions concerning how this stressful event has bothered him/her in the past month. In the study, the respondents did not have to describe what type of event they had experienced, just answer yes/no regarding whether they had experienced anything as in the introduction to the questionnaire: *‘a very stressful experience involving actual or threatened death, serious injury, or sexual violence. It could be something that happened to you directly, something you witnessed, or something you learned happened to a close family member or close friend. Some examples are a serious accident; fire; disaster such as a hurricane, tornado, or earthquake; physical or sexual attack or abuse; war; homicide; or suicide’.* The US version 2013 was used [32] as well as the translated Swedish 2014 version [33, 34] depending on the preference of the interviewee.

To test the reliability of the scales in this specific population we calculated Chronbachs alpha scores; 0,904 for BAI, 0,901 for BDI and 0,893 for the PCL.

### Ethical considerations

The study was approved by the Swedish Central Ethical Review Board (ref. no. Ö 25–2013). Ethical considerations with particular respect for the vulnerable situation of undocumented people were in focus in the study. The ethical principles of informed consent and the possibility of withdrawing consent at any time without any disadvantages for the reporting provider were clearly articulated and repeated. Since many people have experience of traumatic situations, the interview could arouse memories that were difficult to talk about. We therefore placed great emphasis on only using interviewers in the project who had good psychosocial skills and knowledge to handle conversations and meetings with people in vulnerable situations and had information on how to refer the respondent to a counsellor or health staff if it was needed.

### Training of fieldworkers and data collection

Fifteen persons were trained to be interviewers. They had a bachelor’s degree or higher in social science, psychology or medicine. The training took approximately three hours and consisted of information about the study, knowledge about how to use the iPad/tablet for data collection where the questionnaire was placed and how to use the psychiatric instruments. Ethical aspects of performing interviews and interview techniques was also presented. After this the interviewers had support from the principal investigator (HA) and project coordinator (LA) who also performed interviews. Most interviews were conducted with the assistance of authorised translators.

### Statistical analysis

Descriptive analyses with chi-2 analyses were performed with the dichotomised outcomes of the three scales according to the cut-off points defined in the validity studies. Outcomes were analysed by gender as well as overall. The depression and anxiety scales, but not the PCL-5 scale, were found to have a fairly normal distribution, and the variance of these scales was therefore explored in linear regression models. The models included various potential determinants of mental health problems: gender, age, time living as a UM, unstable housing situation, food shortage and having been persecuted or exposed to war in the country of origin.

## Results

### Description of the study population

As can be seen in Table 1, the men in the sample were younger than the women. By analogy with the age distribution, more men were single/widowed (63%) than women (44%). Women more often had children in Sweden (84%) than men (23%). The most common country/region of origin was Afghanistan (28%) and the Middle East (18%). Gender proportions differed widely by country/region of origin; from Afghanistan the majority were men (89%), while there were 63% women from the Middle East.

**Table 1:** Description of the study population: demographic, social and economic factors stratified by gender

	Men		Women		Total	
	N	%	N	%	N	%
Total	48	54.5	40	45.5	88	100
Age						
18–24*	16	33.3	5	12.5	21	22.7
25–39	23	47.9	23	57.5	46	52.3
40+	9	18.8	12	30.0	21	23.9
Country of origin						
Afghanistan	22	45.8	3	7.5	25	28.4
Middle East	7	14.6	9	22.5	16	18.2
Former Yugoslav republics	5	10.4	9	22.5	14	15.9
Former Soviet republics in Asia and Europe	4	8.3	9	22.5	13	14.8
West and Central Africa	3	6.3	1	2.5	4	4.5
East Africa	2	4.2	4	10.0	6	6.8
North Africa	2	4.2	1	2.5	3	3.4
Other	3	6.3	4	10.0	7	8.0
Time as undocumented (years)						
2 years or more	16	33.3	12	30.0	28	31.8
12–24 months	20	41.7	16	40.0	36	40.9
<12 months	12	25.0	12	30.0	24	27.2
Highest completed level of education						
Primary school*	25	52.0	11	27.5	36	40.9
Secondary school	8	16.7	11	27.5	19	21.6
University/college	15	31.2	18	45.0	33	37.5
Civil status						
Single/widowed*	30	62.5	17	43.6	47	54.0
Married/cohabiting/steady relationship*	18	37.5	23	56.4	33	46.0
Parenthood						
Child in Sweden*	11	22.9	33	82.5	42	47.3
Child in another country	6	12.5	0	0	6	5.3
No child*	31	64.6	7	17.5	38	43.2
Having long-standing physical illness	26	55.3	22	56.4	48	55.8
Present housing conditions						
At temporary places, moving around among friends or homeless*	19	39.5	8	25.0	27	30.7
In a fairly stable flat, shared or alone	29	60.5	32	75.0	61	69.3
Has often gone hungry (Yes)	25	52.1	20	50.0	45	56.8
Reasons for leaving home country (More than one option could be given).						
Belonged to a harassed minority	27	56.5	18	45.0	45	51.1
Due to political reasons	23	47.9	18	45.9	41	46.6
There was a war there	23	47.9	14	35.0	37	42.0
Because of religious reasons	17	35.4	11	27.5	28	31.8
Because of social reasons (e.g I was frozen out. I had no one to take care of me)	13	27.1	12	30.0	25	28.4
Because of honour-related problems	13	27.1	11	27.5	24	27.2
Because of economic reasons	14	29.2	7	17.5	21	23.9
Because of disease	7	14.6	3	2.5	10	11.3
None of the above	3	6.2	2	5.0	5	5.7
Because of sexual orientation	1	2.1	1	2.5	2	2.2

* indicates a statistically significant difference between men and women at the *p* < 0.05 level for that row dichotomised

Thirty-two per cent of the sample had been undocumented for more than two years. Women tended to be more highly educated, with 45% having completed university/college vs 31% for men. More men than women reported having insecure housing, either staying in shelters or moving between short-term stays with friends. More than half the studied population stated they were often hungry, and about 56% reported at least one long-standing illness. The most common reasons for leaving the home country and becoming a migrant were belonging to a harassed minority, political factors and war.

### Prevalence of anxiety, depression and PTSD

The prevalence of psychiatric disorders is presented in Table 2. High rates of psychiatric disorders were found without any significant gender differences. A total of 68% of the respondents suffered from either moderate or severe anxiety (total scores 19–63), 71% from either moderate or severe depression (scores 20–63) and 58% fulfilled the criteria for a PTSD diagnosis.

**Table 2:** Psychiatric disorders in undocumented migrants by gender

	Men	Women	All
	*N* = 48	*N* = 40	*N* = 88
	%	%	%
Anxiety (BAI)			
Normal Score 0-9	18.8	5.0	12.5
Mild to Moderate Score 10-18	18.8	20.0	19.3
Moderate to Severe Score 19-29	25.0	35.0	29.5
Severe Score 20-63	37.5	40.0	38.6
	*N* = 38	*N* = 38	*N* = 76
	%	%	%
Depression (BDI)			
Minimial Score 0-13	13.2	15.8	14.5
Mild Score 14-19	15.8	13.2	14.5
Moderate Score 20-28	15.8	10.5	13.2
Severe Score 29-63	55.3	60.5	57.9
	*N* = 32	*N* = 27	*N* = 59
	%	%	%
PTSD (PCL-5)			
No Score 0-38	46.9	37.0	42.4
Yes Score 39-80	53.1	63.0	57.6

### Linear regression models

Results from the linear regression models of the depression and anxiety scales are presented in Table 3. Having an insecure housing situation was a significant risk factor for both anxiety and depression scores. Compared to 18–24 year-old UMs, those aged 25–39 had statistically significantly higher scores on the anxiety scale, while those aged > 40 years had statistically significantly higher scores on the depression scale. Coming from a country at war or having been persecuted also produced the highest effect estimates but did not reach statistical significance in this sample, most likely because only five individuals did not have such a background.

**Table 3:** Linear regression of depression and anxiety scales

	Anxiety (BAI)	Depression (BDI-II)
	*N* = 75	*N* = 75
	B (95% CI)	B (95% CI)
Gender (Female vs male)	4.9 (−1.0–10.8)	−0.3 (−6.7–6.2)
Age 18–24	0 (ref)	0 (ref)
Age 25–39	**7.3 (0.2–14.4)**	5.2 (−2.5–13.0)
Age 40+	8.1 (−0.4–16.8)	**13.6 (4.2–23.0)**
> 2 years undocumented	1.3 (−4.5–7.2)	− 1.2 (−7.6–5.1)
Homeless, no stable housing Yes vs No	**6.6 (0.4–12.8)**	**8.5 (1.7–15.4)**
Often goes hungry	0.7 (−5.0–6.5)	− 1.9 (−8.2–4.4)
Persecution and/or war in country of origin Yes vs No	8.2 (−3.1–19.6)	11.8 (− 0.9. -24.5)

Bold text indicates a statistically significant difference at the p < 0.05-level

## Discussion

In this study of 104 UMs, 88 persons answered questions about mental health. We found alarmingly high rates of depression, anxiety and PTSD with similar prevalences among men and women. Living conditions were precarious overall, and an many reported harassment, political reasons or war as an important reason for leaving the country of origin. An unstable housing situation was identified as a major risk factor for poor mental health.

Our study showed very high prevalences of depression. The prevalence of moderate depression was 13% and that of severe depression as high as 58%. These figures can be compared to findings from a population-based Swedish study from 2009 by Johansson et al. [35], where 11% of the adult Swedish population reported clinically significant depression and 5% major depression (Patient Health questionnaire Depression Scale PHQ-9). Other findings on general population samples in the United States [36] and Norway [37] show prevalences of approximately 7% in the populations suffering from depression within the last 12 months. Myhrvold et al. [38] found in a Norwegian clinical study that 87% of UM patients were suffering from symptoms of major depression (Hopkins Symptom Checklist).

We also found that 30% suffered from moderate to severe anxiety and 39% from severe anxiety. This is much higher than the Swedish general population, where 15% reported clinically significant anxiety (measured on the Generalised Anxiety Disorder Scale). Point prevalence was 9% [35]. Our rates are in closer agreement with the Norwegian study, in which 87% of UM suffered from symptoms of anxiety [38] and are similar to the first study on mental health among UMs in Sweden 2005 where 23 UMs were interviewed and 57% had depression and 48% anxiety (Hopkins Symptom Checklist-25) [23]. These findings suggest that living as a UM implies severe stress and constant worries, which have a profound impact on mental health. The burden of depression and anxiety in our study increased with older age, suggesting that young UMs were somewhat more resilient in this stressful situation. Rates of poor mental health were, however, also high in the youngest age group, and this resilience should therefore not be overestimated.

PTSD prevalence (58%) was much higher in our sample than in both a systematic review of unselected refugee populations (resettled refugees with a residence permit) in Western countries, in which Fazel et al. [39] found that 9% had PTSD and in German study by Fuhrer et al. [40] with 214 adult asylum seekers 18% had PTSD (Harvard Trauma Questionaire). This suggests that for PTSD to be overcome it is crucial that the person feels safe and secure in a long-term perspective, and access to care and counselling is crucial. For UMs this state is never present. Instead they live with fear and insecurity in dealing with daily events and constant stress. Thus, for resettled migrants healing has been possible with successful integration.

Except for the stress-related factors previously mentioned, adverse social and economic conditions increase the risk of developing an illness but may also hamper recovery from existing illness. Our study showed both high rates of mental health problems and harsh living conditions. Having an unstable housing situation was associated with particularly high scores on both the anxiety and depression scales, with 31% living in temporary places or shelters. It seems likely, however, that this is just the heaviest of risk factors in a cumulative load of daily stressors and fears that create the context for this very poor mental health situation. Homelessness was also associated with anxiety and depression in another study [38]. It was also striking that 57% of the UM in our sample stated that they were often hungry and that churches and voluntary organisations were important in providing food as well as meeting basic social needs such as clothes and items necessary to survive the day. Half of respondents in our sample also had children in Sweden to take care of, and many depended on the charity to make ends meet.

Although UMs endure harsh conditions and the majority state that their health has deteriorated while being undocumented, they had still chosen to stay in Sweden. There were many reasons for this: they feared returning to their country of origin since there was a war going on there, they feared going back for political reasons, for religious reasons, or they belonged to a minority and feared harassment. For many it was better to stay and apply for asylum again after four years when rejection decisions expire in Sweden or to move somewhere else than to travel back to the country of origin due to a fear of what they would encounter there.

### Strengths and limitations of the study

This study of UMs has used validated methods to estimate major stress-related psychiatric disorders. Due to the difficulty of reaching this population, we had to use a convenience sample recruited through networks of people helping UMs in the three largest cities in Sweden. Using a convenience sample implies an uncertainty of whether our sample is representative of the whole population of UM’s in Sweden at the time of the study. On the other hand, the definition in itself of the undocumented migrant population excludes the possibility of establishing a “whole” or “representative” population. Since undocumented migrants on well-founded grounds could be expected to be particularly vulnerable and excluded, research using the best possible methods that the circumstances give are warranted. It is possible that this sampling method oversampled UMs with a refugee background, but former asylum-seekers do make up a large proportion of UMs in the Scandinavian countries. It also possible that UMs with a more ordered situation may be less inclined to contact these help networks, thus leading to an overestimation of the true prevalence rates among UMs. Furthermore, although this study used methods that have been validated in several cultural contexts, we cannot guarantee cross-cultural validity for the measures for all participants in this extremely culturally diverse sample. Furthermore, recall bias may be present, especially among those who suffered from PTSD. Interview bias may also influence the results since we had several interviewers and translators. Despite all these obvious methodological problems, there is no reason to believe that the very high prevalences of depression, anxiety and PTSD found in this study are merely artefacts; our results indicate very high rates of poor mental health among UMs.

## Conclusions

When human rights to reasonable living conditions, such as food, housing and safety are severely abused, severe mental health problems are the consequence. This study presents current findings on mental illness among UMs in Sweden and indicates a great need for care and counselling. Furthermore, Sweden has ratified the International Covenant on Economic, Social and Cultural Rights [25] and the Charter of Fundamental Rights of the European Union, and has thus accepted that all individuals have a right to health. This also implies the right to basic necessities for life such as food, housing and access to health care. This study shows that there is a high burden of illness among UM in Sweden, illness that is related to the social determinants of health. Sweden is responsible for fulfilling its obligations towards these human rights: to respect, protect, and fulfil these obligations towards all people staying in the country.
